# A Machine Learning-Based Clustering Using Radiomics of F-18 Fluorodeoxyglucose Positron Emission Tomography/Computed Tomography for the Prediction of Prognosis in Patients with Intrahepatic Cholangiocarcinoma

**DOI:** 10.3390/diagnostics14192245

**Published:** 2024-10-08

**Authors:** Rosie Kwon, Hannah Kim, Keun Soo Ahn, Bong-Il Song, Jinny Lee, Hae Won Kim, Kyoung Sook Won, Hye Won Lee, Tae-Seok Kim, Yonghoon Kim, Koo Jeong Kang

**Affiliations:** 1 Department of Nuclear Medicine, Keimyung University Dongsan Hospital, Keimyung University School of Medicine, Daegu 42601, Republic of Korea; 2 Department of Biology, Columbia University, New York, NY 10027, USA; 3 Department of Surgery, Keimyung University Dongsan Hospital, Keimyung University School of Medicine, Daegu 42601, Republic of Korea; 4 Institute for Cancer Research, Keimyung University, Daegu 42601, Republic of Korea; 5 Department of Medical Information, Keimyung University School of Medicine, Daegu 42601, Republic of Korea; 6 Department of Pathology, Keimyung University Dongsan Hospital, Keimyung University School of Medicine, Daegu 42601, Republic of Korea

**Keywords:** intrahepatic cholangiocarcinoma, clustering, prognosis, survival, F-18 FDG PET/CT

## Abstract

Background: Intrahepatic cholangiocarcinoma (IHCC) is highly aggressive primary hepatic malignancy with an increasing incidence. Objective: This study aimed to develop machine learning-based radiomic clustering using F-18 fluorodeoxyglucose (FDG) positron emission tomography/computed tomography (PET/CT) for predicting recurrence-free survival (RFS) and overall survival (OS) in IHCC. Methods: We retrospectively reviewed pretreatment F-18 FDG PET/CT scans of 60 IHCC patients who underwent surgery without neoadjuvant treatment between January 2008 and July 2020. Radiomic features such as first order, shape, and gray level were extracted from the scans of 52 patients and analyzed using unsupervised hierarchical clustering. Results: Of the 60 patients, 36 experienced recurrence and 31 died during follow-up. Eight patients with a negative FDG uptake were classified as Group 0. The unsupervised hierarchical clustering analysis divided the total cohort into three clusters (Group 1: *n* = 27; Group 2: *n* = 23; Group 3: *n* = 2). The Kaplan–Meier curves showed significant differences in RFS and OS among the clusters (*p* < 0.0001). Multivariate analyses showed that the PET radiomics grouping was an independent prognostic factor for RFS (hazard ratio (HR) = 3.03, *p* = 0.001) and OS (HR = 2.39, *p* = 0.030). Oxidative phosphorylation was significantly activated in Group 1, and the KRAS, P53, and WNT β-catenin pathways were enriched in Group 2. Conclusions: This study demonstrated that machine learning-based PET radiomics clustering can preoperatively predict prognosis and provide valuable information complementing the genomic profiling of IHCC.

## 1. Introduction

Intrahepatic cholangiocarcinoma (IHCC) is one of the aggressive subtypes of cholangiocarcinoma originating from the intrahepatic duct’s epithelial cells [1,2]. Its incidence and mortality have increased globally over recent years [3]. Although surgical resection is the most effective curative treatment leading to prolonged survival for patients with IHCC, fewer than 40% of patients are resectable at diagnosis [4]. Additionally, the 5-year survival rate following resection of IHCC is 20–35% [5], and recurrence is observed in about two-thirds of patients [6]. Consequently, identifying high-risk patients before surgery is paramount to select an effective treatment method. Employing a multidisciplinary approach has become critical in determining the optimal treatment strategy.

IHCC develops through mechanisms driven by chronic inflammation and cholestasis, which lead to molecular changes that promote tumor growth. Chronic inflammation causes DNA damage and activates oncogenic pathways [7], while cholestasis triggers bile acid accumulation and proliferative signaling via ERK1/2, Akt, and NF-κB [8]. Genetic mutations, epigenetic alterations, and changes in the tumor microenvironment further contribute to tumor development [8,9]. These factors often induce both chronic inflammation and cholestasis, highlighting their central role in the progression of IHCC. Understanding these mechanisms is crucial for improving diagnostic and therapeutic strategies.

Radiomics is a non-invasive approach that assesses useful radiographic phenotypes for the prediction of clinical outcomes, and it can be extracted from medical images, such as computed tomography (CT) and magnetic resonance imaging (MRI), through subsequent machine learning analysis [10]. Compared with CT and MRI, the sensitivity of F-18 fluorodeoxyglucose positron emission tomography/computed tomography (F-18 FDG PET/CT) for the detection of IHCCs of >1 cm diameter has been reported to be 85–95%, with a sensitivity of 100% [11]. With its high specificity and sensitivity, F-18 FDG PET/CT-based radiomics may introduce a powerful methodological approach for improving the predictive accuracy of IHCC before curative resection.

Recently, several studies have used F-18 FDG PET radiomics to predict tumors’ characteristics [12,13], lymph node metastasis [14], and prognosis [15,16]. Previous studies utilizing PET radiomics have used supervised learning methods to predict clinical parameters or prognosis [17,18]. However, these models required a larger sample size for training and validation to ensure adequate statistical power for the identification of relevant features associated with the studied clinical outcome [19]. The application of unsupervised hierarchical clustering (UHC) may aid in discovering the inherent structures and relationships within the radiomics data, complementing the shortcomings of the supervised machine learning methods [20]. Unsupervised learning works with unlabeled data, aiming to uncover hidden patterns or structures within the data. It is often used for clustering, reducing dimensionality, and detecting anomalies, with minimal human involvement in data preparation. While supervised learning has a straightforward evaluation due to labeled data, unsupervised learning is more flexible and allows the discovery of unknown patterns.

Therefore, this study aimed to use radiomic features derived from F-18 FDG PET/CT images with unsupervised machine learning algorithms to build a model that could accurately predict recurrence-free survival (RFS) and overall survival (OS) in IHCC preoperatively. Gene expression data (RNA sequencing data using the next-generation sequencing method) and the clinical parameters of IHCC patients were used to identify candidate biomarkers based on the correlations between the genes and radiomic subgroups.

## 2. Materials and Methods

### 2.1. Patients, Tissue Samples, and Clinical Analysis

**Figure 1:**
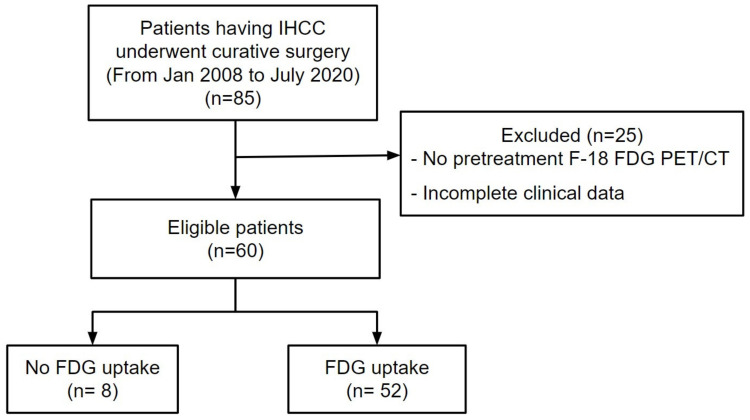
Flow diagram of patient selection. Of the 85 patients who received curative surgery for the IHCC treatment, 25 patients were excluded. Finally, 60 patients were enrolled in this study.

The institutional review board of Keimyung University Dongsan Hospital, Daegu, Republic of Korea (IRB Number 2021-11-009) approved the present trial. The need for informed consent was waived due to the retrospective nature of the present study, and all data were anonymized prior to the analysis. In total, 85 consecutive patients with IHCC who had undergone curative surgery between January 2008 and July 2020 at Keimyung University Dongsan Hospital were identified. The inclusion criteria were: (1) the patient underwent tumor resection for pathologically diagnosed IHCC, (2) no other treatment prior to surgery, and (3) available clinical data and surgical records. The exclusion criteria were as follows: (1) no F-18 FDG PET/CT scan prior to the operation and (2) incomplete clinical data. The primary clinical data included carcinoembryonic antigen (CEA), carbohydrate antigen 19-9 (CA19-9), age, gross and microscopic pathologic types, tumor size, American Joint Committee on Cancer stage, maximum standardized uptake values (SUVmax), and metabolic tumor volume (MTV). On the basis of the inclusion and exclusion criteria, 85 patients were enrolled and 25 patients were excluded in this study. Therefore, in total, 60 IHCC patients were included during the study period above. The demographics, preoperative serum biomarkers, pathologic characteristics, recurrence, and survival outcomes of the patients were analyzed. A flow chart of the patient selection process is shown in Figure 1.

### 2.2. F-18 FDG PET/CT Image Acquisition

F-18 FDG PET/CT scans were obtained prior to surgical resection. All patients were instructed to fast for 6 h, and their blood glucose levels were checked to ensure the level was less than 150 mg/dL prior to the administration of F-18 FDG. The whole scan was performed 60 min after intravenously administering 5.5 MBq/kg of F-18 FDG. Two PET/CT scanners (Siemens Healthineers Knoxville, TN, USA Discovery STE; GE Healthcare, Milwaukee, WI, United States or Biograph mCT) were used to acquire the images. Low-dose CT scans (Discovery STe; peak voltage, 120 kV; automated tube current, 60–150 mA; and slice thickness, 3.75 mm, Biograph mCT; peak voltage, 120 kV, automated exposure control using CARE Dose4D; slice thickness, 3 mm) were obtained for correction of attenuation and anatomical localization. The PET images were acquired at 3 min per bed position in 3-dimensional mode immediately after the CT scan.

The PET images were converted into SUV units by normalizing the radioactivity concentration to the injected dosage of F-18 FDG and the patient’s body weight. The SUVmax was calculated using the attenuation-corrected images, the body weight of the patient, and the amount of F-18 FDG; the equation of SUVmax can be written as follows: SUVmax = maximum activity (kBq) (injected activity (kBq)/bodyweight (g)).

### 2.3. Image Processing and Analysis

**Figure 2:**
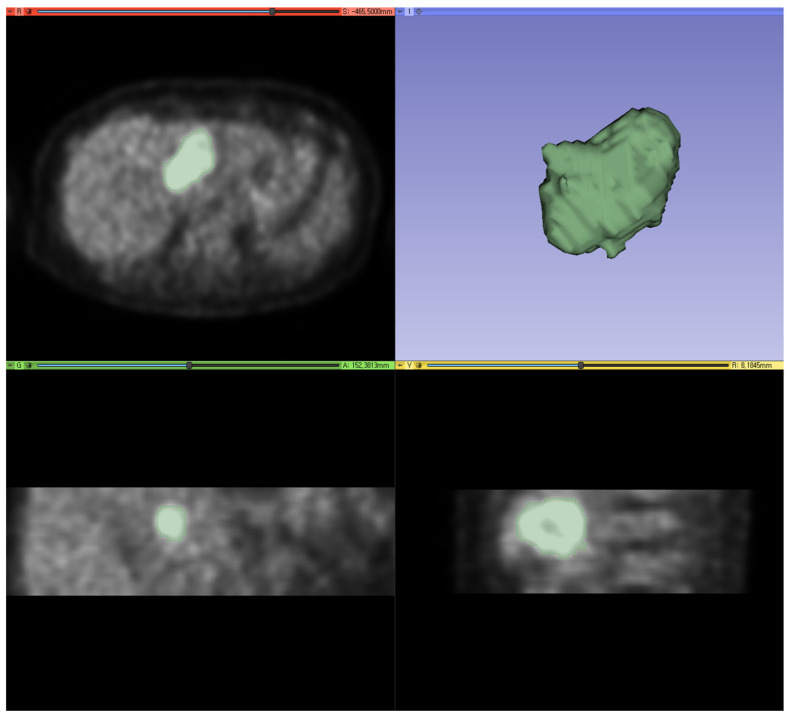
An exemplary case of tumor segmentation. Using the maximum entropy method, segmentation of the IHCC was performed with 3D Slicer software (version 5.2.1).

IHCC PET images were formatted as digital imaging and communications in medicine (DICOM) files, and we cropped the areas with myocardial or urinary high FDG activities, which may have interfered with the segmentation of the IHCC [21]. Segmentation of the IHCC was performed with 3D Slicer software (version 5.2.1) using the maximum entropy tool, a semi-automatic segmentation method, to minimize intra- and interobserver variability [22]. Voxels with intensities above the maximum entropy were classified as IHCC, and those below the maximum entropy were considered as the background (Figure 2).

The images were reconstructed with a 3 mm × 3 mm × 3 mm voxel size prior to the radiomic computation, and voxel intensities were resampled into equally spaced bins using a bin width of 0.1 SUV units to reduce the images’ noise and normalize the intensities of all patients. Radiomic features from PET imaging data were extracted using the PyRadiomics package in Python [23]. In total, 960 quantitative imaging features were included in the analysis and normalized using a z-score, where each feature was normalized as z = (x − μ)/σ, where x, μ, and σ are the feature, the mean, and the standard deviation of the features, respectively [24].

We performed balanced iterative reducing and clustering using hierarchies (BIRCH), an unsupervised clustering algorithm that efficiently clusters large datasets by working on the entire dataset and generating a compact hierarchical structure, based on the radiomic features [25].

**Figure 3:**
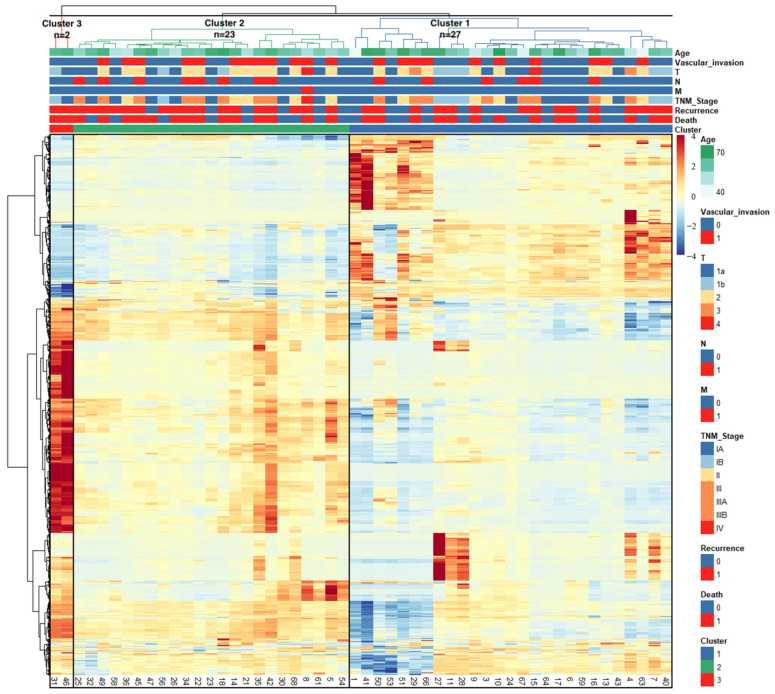
Heatmap depicting the correlations between the patients’ characteristics and radiomics. Patient IDs are represented by columns, and radiomic features are represented by rows in the matrix.

The Pheatmap package in R software (version 4.1.1, https://www.r-project.org, accessed on 10 August 2021) was utilized to visualize the association of F-18 FDG PET/CT radiomics with clinical information (Figure 3). The web-based IHCC classification calculator was developed by Streamlit [26].

### 2.4. Identification of Differentially Expressed Genes and Activated Pathways

For 19 patients of the present study’s population, RNA expression data were available in GSE107943 [27], which consisted of 30 IHCC surgical specimens and 28 non-cancerous surrounding liver specimens. Differentially expressed genes (DEGs) were identified to discover genes that showed significant differences in their expression between Clusters 1 and 2 using the EdgeR package in R software (4.1.1), and gene set enrichment analysis (GSEA) was conducted to investigate the activated pathways [28,29].

### 2.5. Statistical Analysis

RFS was determined as the time from the date of surgery to the first evidence of recurrent disease or the last follow-up, whichever occurred first. OS was determined as the time from the date of surgery to the date of death or last follow-up. To compare the survival probabilities from the Kaplan–Meier estimates of each group, a log-rank test was applied, enabling us to make inferences about the probability of survival. The hazard ratio (HR) with a 95% confidence interval (CI) for each parameter was estimated by univariate and multivariate Cox proportional hazard regression analyses. Statistical analysis was conducted using MedCalc version 18.10.2 (MedCalc Software, Ostend, Belgium). A *p* value less than 0.05 was considered to be statistically significant.

## 3. Results

### 3.1. Patient Characteristics

In total, 60 patients with IHCC who satisfied the study criteria were included in the study population. The average age of women was 64 years (range: 38 to 75 years old), and the average age of men was 56 years (range: 39 to 81 years old). The average follow-up time was 27 months (range: 0 to 168 months). In total, 36 (60%) patients experienced recurrence, and 31 (51.7%) patients died before the last follow-up date.

### 3.2. Radiomics-Based Clustering of Patients

The BIRCH analysis was conducted using 960 radiomic features based on F-18 FDG PET images and grouped patients with similar features in the images. However, eight patients (13.3%) who did not have F-18 FDG uptake were classified as Cluster 0, and their data could not undergo clustering analysis.

The heatmap with dendrograms illustrates the correlation matrix of 52 patients with positive FDG uptake and radiomics. The optimal number of clusters was determined by the elbow curve method (k = 3), allocating 27 patients (45%) to Cluster 1, 23 patients (38.3%) to Cluster 2, and 2 patients (3.3%) to Cluster 3 (Figure 3). The baseline demographics and clinical characteristics of the patients, stratified by subgroups, are summarized in Table 1.

### 3.3. Survival Analysis

**Figure 4:**
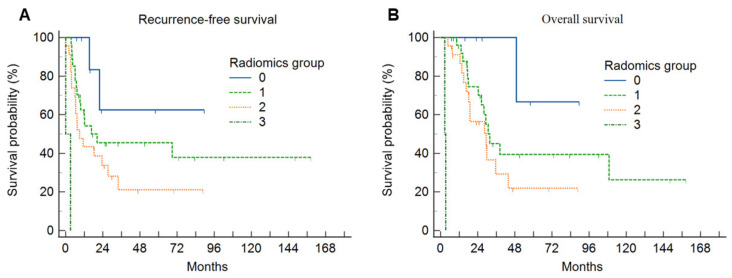
(**A**) Cumulative recurrence-free survival curves and (**B**) overall survival curves according to the PET radiomics group. The high radiomics group was associated with a significantly lower recurrence-free survival rate and overall survival rate compared with the low radiomics group.

**Figure 5:**
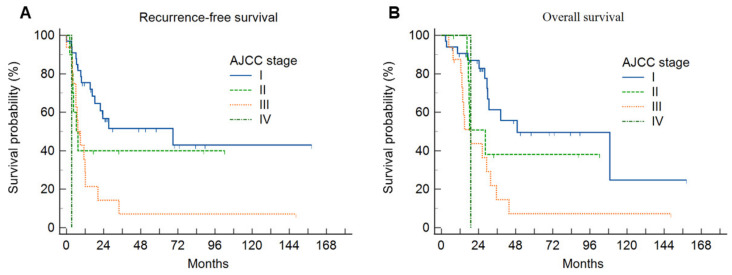
(**A**) Cumulative recurrence-free survival curves and (**B**) overall survival curves according to the AJCC stage. The advanced AJCC state was associated with a significantly lower recurrence-free survival rate and overall survival rate.

The Kaplan–Meier curves demonstrated that there were significant differences in the survival rates (Figure 4 and Figure 5). Statistically significant factors from the univariate analysis were used in the Cox regression multivariate analysis to determine independent predictors of RFS or OS for IHCC patients (Table 2 and Table 3).

The univariate analysis revealed that RFS was significantly correlated with sex, CA 19-9, vascular invasion, pathologic T stage, pathologic N stage, and PET radiomics group. In the multivariate analysis, CA 19-9 (HR 5.50, 95% CI, 2.12–14.25), pathologic T stage (HR 2.31, 95% CI, 1.16–4.61), pathologic N stage (HR 3.15, 95% CI, 1.25–7.93), and PET radiomics group (HR 3.03, 95% CI, 1.55–5.95) were identified as independent prognostic factors for RFS.

Factors associated with an increased risk of OS included CA 19-9, vascular invasion, pathologic T stage, pathologic N stage, and PET radiomics group, as revealed by the univariate analysis. In the multivariate analysis, CA 19-9 (HR 4.87, 95% CI, 1.76–13.44), pathologic N stage (HR 4.09, 95% CI, 1.57–10.70), and PET radiomics group (HR 2.39, 95% CI, 1.09–5.25) were significantly related to OS. The web-based IHCC classification calculator is available at https://ihccbysong.streamlit.app/ (accessed on 15 August 2023. username: KUDH; password: Song).

### 3.4. RNA Expression Profiling: Analysis of DEGs and Activated Pathways

In total, 42 DEGs with a log fold change greater than 1.0 or less than −1.0 between Clusters 1 and 2 and a false discovery rate less than 0.05 were selected (Supplementary Table S1). Collagen Type XI Alpha 2 Chain (COL11A2) was significantly upregulated in Cluster 1, while 41 genes were upregulated in Cluster 2.

**Figure 6:**
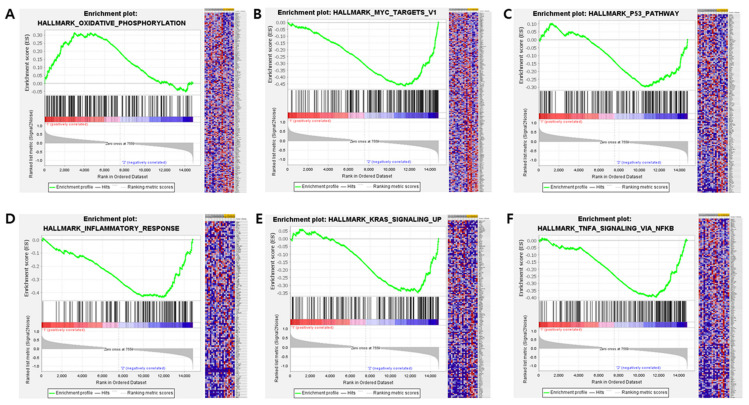
Gene set enrichment analysis, showing significantly activated oxidative phosphorylation in Cluster 1 (**A**) and significantly activated MYC targets v1 (**B**), the p53 pathway (**C**), the inflammatory response (**D**), KRAS signaling (**E**), and TNF alpha signaling via NF-κB (**F**) in Cluster 2.

Pathway analysis showed that signaling related to oxidative phosphorylation was significantly activated in Cluster 1 (false discovery rate < 0.25), and the KRAS, P53 and WNT β-catenin pathways were enriched in Cluster 2. Many of the significant gene sets or pathways were related to inflammatory and immune responses, including the interferon alpha response, IL-2 and cytokine-mediated signaling pathways (Figure 6, Supplementary Table S2).

## 4. Discussion

In this study, we extracted radiomic, SUVmax, and MTV data from F-18 FDG PET/CT images of 60 IHCC patients. All IHCC patients were classified into four groups, with eight patients without FDG uptake as Group 0, and three clusters of patients with distinct radiomic patterns generated by the UHC. Groups 0, 1, 2, and 3 were classified as low-risk, intermediate-risk, high-risk, and extremely high-risk groups, respectively. The difference in the survival probabilities of the four groups was statistically significant, with Group 0 having the best prognosis and Group 3 having the worst.

Several studies have shown that high FDG uptake by the primary tumor is associated with a high tumor grade [30], vascular invasion [31,32], lymph node metastasis [33,34,35], and poor prognosis [36,37,38]. Zhang et al. revealed that the SUVmax of the primary tumor was significantly higher in poorly differentiated groups, larger tumors, and high Ki67 expression groups in IHCC patients [30]. Jiang et al. proposed the use of radiomic analysis for classifying and predicting microvascular invasion in IHCC [32]. Moreover, Fiz et al. showed that PET-based radiomics of IHCC can predict the pathology data and allow a reliable preoperative evaluation of the prognosis [16]. In line with these findings, our study demonstrated that patients with a negative FDG uptake (Group 0) had no vascular invasion or tumor lymph node metastasis and had the best prognosis, and patients with a positive FDG uptake classified by the UHC of PET radiomics had a significantly worse prognosis and a different genomic profile compared with patients with a negative FDG uptake.

In the present study, Cluster 2 (the high-risk group) exhibited enrichments in oncogenic signaling pathways including the KRAS, p53, TNF alpha, and WNT β-catenin pathways. These pathways, known as poor prognosis factors in cholangiocarcinoma and other cancers, activate oncogenic signaling or the suppression of tumor suppression function [27,39,40,41,42]. This result supports the findings from the survival analyses, in which the prognosis of Cluster 2 was significantly poorer than that of Cluster 1. The consistency across data indicated that clustering by radiomics represents genomic features, although genetic data were only available for a subset of the patients. The relevance between radiomics and genomics may suggest the possibility of predicting genetic characteristics utilizing radiomic analysis.

Accurate and consistent segmentation is essential for radiomic analysis to prevent incorrect feature extraction, ensuring the reproducibility and reliability of results. In this study, the maximum entropy segmentation method was employed for tumor segmentation because the fixed absolute threshold of SUV could not properly segment the tumor due to the high background activity of the liver, and the fixed relative threshold method resulted in underestimated tumor segmentation with a significantly higher SUVmax. The maximum entropy segmentation principle determines the optimal threshold value by analyzing the intensity histogram. As a semi-automatic tool, it minimizes the need for user input, such as defining the regions of interest, effectively reducing intra- and interobserver variability.

The results of this study suggest that radiomics can preoperatively predict prognosis and personalize treatment and management for IHCC patients by reducing sample error in biopsies through its non-invasive and reproducible imaging [43]. In addition, radiomics can serve as a tool to provide insights into the testing of specific genes’ expression in biopsy material or the optimal location of the biopsy site [44]. This information can be used to improve the accuracy of cancer diagnosis, predict the disease’s progression and response to treatment, and ultimately guide treatment decisions. By identifying specific genetic alterations that are associated with certain imaging features, we can gain insights into the underlying biological mechanisms driving cancer’s growth and identify potential drug targets. In this study, there were no clear clinical and pathological differences among the four radiomics-based groups. It was also found to be a significant factor of recurrence and survival, along with pathologic staging. This shows that radiomics independently reflects the genetic characteristics of IHCC patients, regardless of the clinicopathological findings, and are valuable in determining the severity and prognosis of IHCC. This information can be used to develop new targeted therapies or repurpose existing drugs for more effective cancer treatment. Overall, the cross-validation of F-18 FDG PET-based radiomics with genomics holds promise for more accurate predictions of prognosis and more personalized management of IHCC patients.

In this study, the use of unsupervised learning techniques was driven by the limited availability of data. Unlike supervised methods, which can overfit small datasets, unsupervised learning identifies hidden patterns and clinically relevant subgroups without the need for labeled data. Clustering revealed distinct groups with different prognoses, offering insights into variability in the RFS and OS outcomes despite the small cohort. While these findings are an important first step in understanding IHCC’s heterogeneity, larger studies are needed to validate the results. Future research should incorporate larger datasets, possibly from multiple centers, to improve the robustness of predictive models for IHCC.

KRAS and TP53 mutations are associated with poor prognosis and a high tumor mutation burden in IHCC [45]. These mutations, along with activation of the WNT/β-catenin pathway, correlate with unfavorable outcomes, such as reduced survival in IHCC patients [46]. The oncogenic effects of KRAS and TP53, combined with alterations of the WNT pathway, highlight their role in driving tumor progression. While radiomic features can be associated with and potentially predict certain gene expression patterns in cancer [47,48], the relationship is complex. Integrating radiomic and genomic data shows promise for improving the characterization of cancer and predicted prognosis, but more research is needed to fully understand and validate these associations.

There were some limitations to this study. First, the current study was a single-center retrospective study, which could have been influenced by selection bias. To make the findings more generalizable, performing prospective studies is recommended to confirm the prognostic impact of UHC using PET radiomics in patients with IHCC. Second, there is a potential risk of sampling bias, as genomic data were only available for a subset of patients. Lastly, external validation was not performed due to the limited number of patients. To overcome this issue, genetic analysis was integrated to provide a more comprehensive understanding of the characteristics of the radiomics clusters. More diverse datasets and further external validation are required to develop and verify the performance of the radiomic clustering model.

## 5. Conclusions

F-18 FDG PET findings and UHC using PET radiomics showed the feasibility of predicting RFS and OS for patients with IHCC. This study demonstrated the potential of PET radiomics to provide valuable information complementary to the genomic profiling of IHCC, accurately and preoperatively predict prognosis, and guide personalized treatment strategies.

## Figures and Tables

**Table 1:** Patient characteristics.

Characteristics	Group 0 (*n* = 8)	Group 1 (*n* = 27)	Group 2 (*n* = 23)	Group 3 (*n* = 2)	*p*
Age	67.0 ± 8.8	62.7 ± 11.9	62.4 ± 7.8	69.0 ± 1.4	0.576
Sex					0.175
Female	2 (25.0%)	10 (37.0%)	6 (26.1%)	2 (100.0%)	
Male	6 (75.0%)	17 (63.0%)	17 (73.9%)	0 (0.0%)	
CEA	3.6 ± 2.2	2.3 ± 2.8	3.5 ± 1.6	2.4 ± 2.7	0.494
CA19-9	295.8 ± 258.7	417.1 ± 1286.4	554.1 ± 1727.4	42.4 ± 18.4	0.955
Differentiation					0.667
Well	0 (0.0%)	1 (3.7%)	0 (0.0%)	0 (0.0%)	
Moderately	7 (87.5%)	15 (55.6%)	16 (69.6%)	1 (50.0%)	
Poorly	1 (12.5%)	11 (40.7%)	7 (30.4%)	1 (50.0%)	
Vascular invasion					0.043
Negative	8 (100.0%)	17 (63.0%)	11 (47.8%)	2 (100.0%)	
Positive	0 (0.0%)	10 (37.0%)	12 (52.2%)	0 (0.0%)	
Gross appearance					0.388
Mass forming	6 (75.0%)	25 (92.6%)	18 (78.3%)	2 (100.0%)	
Periductal infiltrating or mixed	2 (25.0%)	2 (7.4%)	5 (21.7%)	0 (0.0%)	
Microscopic type *					0.827
Small duct type	4 (57.1%)	14 (56.0%)	10 (47.6%)	0 (0.0%)	
Large duct type	3 (42.9%)	11 (44.0%)	11 (52.4%)	1 (100.0%)	
Tumor size (cm)	2.2 ± 0.9	5.6 ± 2.9	4.6 ± 1.5	4.3 ± 3.1	0.006
LN metastasis					0.154
Negative	8 (100.0%)	22 (81.5%)	15 (65.2%)	2 (100.0%)	
Positive	0 (0.0%)	5 (18.5%)	8 (34.8%)	0 (0.0%)	
Pathologic T stage					0.146
1a	8 (100.0%)	11 (40.7%)	9 (39.1%)	1 (50.0%)	
1b	0 (0.0%)	5 (18.5%)	2 (8.7%)	1 (50.0%)	
2	0 (0.0%)	8 (29.6%)	11 (47.8%)	0 (0.0%)	
3	0 (0.0%)	2 (7.4%)	0 (0.0%)	0 (0.0%)	
4	0 (0.0%)	1 (3.7%)	1 (4.3%)	0 (0.0%)	
Pathologic N stage					0.191
0	8 (100.0%)	21 (77.8%)	15 (65.2%)	2 (100.0%)	
1	0 (0.0%)	6 (22.2%)	8 (34.8%)	0 (0.0%)	
Pathologic M stage					0.651
0	8 (100.0%)	27 (100.0%)	22 (95.7%)	2 (100.0%)	
1	0 (0.0%)	0 (0.0%)	1 (4.3%)	0 (0.0%)	
8th AJCC TNM stage					0.127
IA	8 (100.0%)	10 (37.0%)	7 (30.4%)	0 (0.0%)	
IB	0 (0.0%)	5 (18.5%)	1 (4.3%)	1 (50.0%)	
II	0 (0.0%)	5 (18.5%)	6 (26.1%)	1 (50.0%)	
III	0 (0.0%)	1 (3.7%)	0 (0.0%)	0 (0.0%)	
IIIA	0 (0.0%)	1 (3.7%)	0 (0.0%)	0 (0.0%)	
IIIB	0 (0.0%)	5 (18.5%)	8 (34.8%)	0 (0.0%)	
IV	0 (0.0%)	0 (0.0%)	1 (4.3%)	0 (0.0%)	
SUVmax	-	6.8 ± 1.8	11.6 ± 2.8	28.8 ± 5.6	0
MTV (mm^3^)	-	76,508.0 ± 135,011.5	37,964.3 ± 37,340.1	99,738.0 ± 12,982.5	0.357

The data are presented as the mean ± standard deviation. CEA, carcinoembryonic antigen; CA19-9, carbohydrate antigen 19-9; LN, lymph node; AJCC, American Joint Committee on Cancer; TNM, tumor node metastasis; SUVmax, maximum standardized uptake value; MTV, metabolic tumor volume. * Six patients had mixed microscopic types, and we excluded them from the microscopic classification.

**Table 2:** Univariate and multivariate analyses of the prognostic factors of recurrence-free survival.

	Univariate Analysis		Multivariate Analysis	
Variables	HR (95% CI)	*p* Value	HR (95% CI)	*p* Value
Age, years (<65 vs. ≥65)	1.05 (0.55–2.02)	0.886		
Sex (female vs. male)	2.28 (1.03–5.04)	0.043	0.69 (0.26–1.82)	0.453
CEA (<5 ng/mL vs. ≥5 ng/mL)	0.92 (0.31–2.70)	0.879		
CA 19-9 (<37 U/mL vs. ≥37 U/mL)	2.57 (1.24–5.32)	0.011	5.50 (2.12–14.25)	<0.001
Tumor differentiation (well, moderately, poorly)	1.77 (0.94–3.33)	0.079		
Vascular invasion (No vs. Yes)	2.54 (1.31–4.95)	0.006	0.35 (0.10–1.27)	0.111
Tumor size (<5 cm vs. ≥5 cm)	1.46 (0.75–2.85)	0.262		
Pathologic T stage (T1, T2, T3, T4)	1.79 (1.24–2.59)	0.002	2.31 (1.16–4.61)	0.018
Pathologic N stage (N0, N1)	2.45 (1.21–4.96)	0.013	3.15 (1.25–7.93)	0.015
PET radiomics group (0, 1, 2, 3)	2.19 (1.28–3.47)	0.004	3.03 (1.55–5.95)	0.001

CEA, carcinoembryonic antigen; CA19-9, carbohydrate antigen 19-9; HR, hazard ratio; CI, confidence interval.

**Table 3:** Univariate and multivariate analyses of the prognostic factors of overall survival.

	Univariate Analysis		Multivariate Analysis	
Variables	HR (95% CI)	*p* Value	HR (95% CI)	*p* Value
Age, years (<65 vs. ≥65)	1.54 (0.75–3.19)	0.242		
Sex (female vs. male)	1.89 (0.86–4.13)	0.111		
CEA (<5 ng/mL vs. ≥5 ng/mL)	0.61 (0.14–2.66)	0.514		
CA 19-9 (<37 U/mL vs. ≥37 U/mL)	2.72 (1.25–5.93)	0.012	4.87 (1.76–13.44)	0.002
Tumor differentiation (well, moderately, poorly)	1.72 (0.86–3.46)	0.126		
Vascular invasion (no vs. yes)	3.18 (1.53–6.62)	0.002	1.01 (0.30–3.38)	0.985
Tumor size (<5 cm vs. ≥5 cm)	1.26 (0.62–2.57)	0.528		
Pathologic T stage (T1, T2, T3, T4)	2.07 (1.39–3.08)	<0.001	1.55 (0.80–3.03)	0.197
Pathologic N stage (N0, N1)	2.60 (1.24–5.47)	0.012	4.09 (1.57–10.70)	0.004
PET radiomics group (0, 1, 2, 3)	2.46 (1.32–4.56)	0.004	2.39 (1.09–5.25)	0.030

CEA, carcinoembryonic antigen; CA19-9, carbohydrate antigen 19-9; HR, hazard ratio; CI, confidence interval.

## Data Availability

Data supporting the present study are available from the corresponding author upon reasonable request. Publicly available RNA expression datasets were analyzed in this study. This data can be found here: https://www.ncbi.nlm.nih.gov/geo/query/acc.cgi?acc=GSE107943 (accessed on 1 September 2024).

## Supplementary Materials

The following supporting information can be downloaded at: https://www.mdpi.com/article/10.3390/diagnostics14192245/s1, Table S1. Differentially expressed genes between cluster 1 and 2. Table S2. Enriched pathways by GSEA analysis.
