# Supplementary material for: A Machine Learning-Based Clustering Using Radiomics of F-18 Fluorodeoxyglucose Positron Emission Tomography/Computed Tomography for the Prediction of Prognosis in Patients with Intrahepatic Cholangiocarcinoma
Source: Diagnostics (Basel). 2024 Oct 8;14(19):2245. doi: 10.3390/diagnostics14192245 (PMC11475304; doi:10.3390/diagnostics14192245)
Supplement: Supplementary file 1 [file diagnostics-14-02245-s001.zip › diagnostics-3212969-supplementary.pdf]

Table S1. Differentially expressed genes between cluster 1 and 2

Up-regulated cluster	Gene	Log fold change	*P* value	FDR
cluster1	COL11A2	-4.958777097	3.60E-05	0.02006586158
cluster2	AMIGO2	2.826625289	4.30E-05	0.02323873204
cluster2	ANXA1	2.697034346	2.70E-06	0.003443750532
cluster2	APOD	3.522047625	8.96E-06	0.008414166787
cluster2	APOL1	2.125808467	2.23E-05	0.01588448531
cluster2	ASCL2	4.114113779	1.18E-06	0.001913133104
cluster2	CA12	3.361311789	5.90E-08	0.0002104685642
cluster2	CYP4X1	3.32784173	4.87E-07	0.001087033506
cluster2	DCBLD2	2.781371731	2.49E-05	0.01592501133
cluster2	DOK5	2.212875114	1.36E-04	0.04624719152
cluster2	DPEP1	6.085245082	1.72E-08	0.0001538519671
cluster2	DUOX2	4.601920013	1.37E-06	0.001934830434
cluster2	DUOXA1	4.08314746	6.94E-06	0.006875700997
cluster2	DUOXA2	5.252312563	4.84E-08	0.0002104685642
cluster2	F3	4.324832708	7.38E-10	1.32E-05
cluster2	FCGBP	3.038530089	9.43E-06	0.008414166787
cluster2	FOXL1	2.668722128	1.08E-04	0.04681712023
cluster2	GALNT5	4.122694873	1.15E-05	0.009775963389
cluster2	GNA15	2.283394201	3.25E-05	0.01872348081
cluster2	IDO1	3.882274892	2.59E-05	0.01592501133
cluster2	IL1B	3.09903945	1.46E-05	0.01183998464
cluster2	IL20RB	2.201193388	5.94E-05	0.02942381736
cluster2	ITM2A	3.454379134	1.84E-05	0.01370183538
cluster2	KCNJ15	4.169379738	2.31E-07	0.0005884753562
cluster2	KIT	1.894660737	6.47E-05	0.03121453539
cluster2	KRT17	4.47142497	5.71E-06	0.005991000552
cluster2	LGALS2	3.184280116	2.76E-05	0.01644406504
cluster2	LTF	6.01515827	1.00E-06	0.001790694799
cluster2	LYPD3	2.68300604	8.83E-05	0.04042039929
cluster2	MAP3K5	1.713821525	8.46E-05	0.03974217295
cluster2	MEDAG	3.637407588	1.24E-07	0.0003674365967
cluster2	MMP28	4.339870626	4.30E-08	0.0002104685642
cluster2	MUC6	4.061832285	3.17E-06	0.003776339361
cluster2	PLAUR	2.041763707	2.51E-05	0.01592501133
cluster2	PRELID2	1.87483658	1.74E-05	0.01349744227
cluster2	RP11-442H21.2	1.917628504	1.10E-04	0.04681712023
cluster2	S100A2	3.511165771	4.93E-06	0.005494202556
cluster2	S100A9	3.449121993	1.41E-06	0.001934830434
cluster2	SPON1	3.235855113	4.74E-05	0.02415012848
cluster2	SRPX	2.4875592	4.43E-05	0.02323873204
cluster2	SYTL1	2.242798021	2.49E-05	0.01592501133
cluster2	TCN1	4.505720375	6.62E-07	0.001312156454

FDR, false discovery rate

Table S2. Enriched pathways by GSEA analysis

Pathways	SIZE	ES	NES		FDR q-value	LEADING EDGE
<Cluster 1>						
HALLMARK_OXIDATIVE_PHOSPHORYLATION	184	0.314	1.573	1.89E-03	4.56E-02	tags=30%, list=20%, signal=38%
<Cluster 2>						
HALLMARK_MYC_TARGETS_V1	193	-0.466	-2.403	0	0	tags=43%, list=22%, signal=54%
HALLMARK_G2M_CHECKPOINT	168	-0.467	-2.336	0	0	tags=52%, list=29%, signal=73%
HALLMARK_INTERFERON_ALPHA_RESPONSE	93	-0.504	-2.275	0	0	tags=51%, list=26%, signal=68%
HALLMARK_INFLAMMATORY_RESPONSE	140	-0.436	-2.127	0	0	tags=39%, list=20%, signal=48%
HALLMARK_E2F_TARGETS	178	-0.424	-2.124	0	0	tags=48%, list=26%, signal=65%
HALLMARK_INTERFERON_GAMMA_RESPONSE	177	-0.422	-2.099	0	0	tags=44%, list=26%, signal=58%
HALLMARK_TNFA_SIGNALING_VIA_NFKB	183	-0.396	-2.013	0	2.44E-04	tags=39%, list=21%, signal=49%
HALLMARK_HYPOXIA	168	-0.344	-1.697	0	1.06E-02	tags=38%, list=25%, signal=50%
HALLMARK_KRAS_SIGNALING_UP	156	-0.348	-1.704	0	1.08E-02	tags=28%, list=14%, signal=32%
HALLMARK_IL2_STAT5_SIGNALING	153	-0.340	-1.677	0	1.08E-02	tags=33%, list=19%, signal=40%
HALLMARK_MTORC1_SIGNALING	186	-0.328	-1.669	0	1.10E-02	tags=42%, list=29%, signal=59%
HALLMARK_ESTROGEN_RESPONSE_LATE	161	-0.331	-1.648	0	1.12E-02	tags=33%, list=21%, signal=41%
HALLMARK_GLYCOLYSIS	175	-0.315	-1.557	6.29E-03	2.51E-02	tags=36%, list=23%, signal=46%
HALLMARK_IL6_JAK_STAT3_SIGNALING	70	-0.354	-1.538	1.08E-02	2.76E-02	tags=33%, list=18%, signal=40%
HALLMARK_MYC_TARGETS_V2	57	-0.368	-1.521	2.39E-02	2.79E-02	tags=39%, list=24%, signal=51%
HALLMARK_ALLOGRAFT_REJECTION	140	-0.316	-1.524	2.04E-03	2.84E-02	tags=41%, list=26%, signal=54%
HALLMARK_ANDROGEN_RESPONSE	87	-0.332	-1.498	1.27E-02	3.01E-02	tags=41%, list=26%, signal=56%
HALLMARK_P53_PATHWAY	172	-0.298	-1.490	2.07E-03	3.05E-02	tags=44%, list=29%, signal=60%
HALLMARK_COMPLEMENT	160	-0.302	-1.498	2.06E-03	3.19E-02	tags=43%, list=29%, signal=60%
HALLMARK_ESTROGEN_RESPONSE_EARLY	164	-0.285	-1.415	1.52E-02	5.37E-02	tags=21%, list=13%, signal=24%
HALLMARK_CHOLESTEROL_HOMEOSTASIS	69	-0.327	-1.420	3.41E-02	5.41E-02	tags=32%, list=21%, signal=40%
HALLMARK_EPITHELIAL_MESENCHYMAL_TRANSITION	162	-0.278	-1.380	2.32E-02	6.45E-02	tags=38%, list=26%, signal=50%
HALLMARK_WNT_BETA_CATENIN_SIGNALING	35	-0.362	-1.350	6.73E-02	7.79E-02	tags=31%, list=13%, signal=36%
HALLMARK_APOPTOSIS	142	-0.267	-1.306	3.48E-02	1.01E-01	tags=42%, list=28%, signal=57%
HALLMARK_UNFOLDED_PROTEIN_RESPONSE	106	-0.288	-1.312	6.99E-02	1.02E-01	tags=37%, list=27%, signal=50%
HALLMARK_ANGIOGENESIS	35	-0.335	-1.258	1.66E-01	1.38E-01	tags=43%, list=24%, signal=56%
HALLMARK_COAGULATION	123	-0.257	-1.224	1.06E-01	1.64E-01	tags=44%, list=30%, signal=62%
HALLMARK_UV_RESPONSE_UP	132	-0.255	-1.228	1.07E-01	1.66E-01	tags=35%, list=26%, signal=46%
HALLMARK_UV_RESPONSE_DN	114	-0.253	-1.201	1.29E-01	1.88E-01	tags=16%, list=10%, signal=17%
HALLMARK_XENOBIOTIC_METABOLISM	176	-0.233	-1.166	1.27E-01	2.33E-01	tags=35%, list=28%, signal=48%
KEGG_GRAFT_VERSUS_HOST_DISEASE	24	-0.671	-2.257	0	0	tags=75%, list=26%, signal=101%
KEGG_DRUG_METABOLISM_OTHER_ENZYMES	42	-0.548	-2.134	0	3.63E-04	tags=45%, list=12%, signal=51%
KEGG_AUTOIMMUNE_THYROID_DISEASE	24	-0.629	-2.141	0	4.84E-04	tags=71%, list=26%, signal=95%
KEGG_RIBOSOME	84	-0.485	-2.169	0	7.27E-04	tags=56%, list=30%, signal=80%
KEGG_TYPE_I_DIABETES_MELLITUS	27	-0.599	-2.099	0	8.61E-04	tags=63%, list=26%, signal=85%

KEGG_SPLICEOSOME	122	-0.431	-2.058	0	3.45E-03	tags=39%, list=20%, signal=48%
KEGG_ALLOGRAFT_REJECTION	23	-0.624	-2.033	0	3.96E-03	tags=70%, list=26%, signal=94%
KEGG_VIRAL_MYOCARDITIS	50	-0.498	-2.013	0	5.13E-03	tags=52%, list=27%, signal=71%
KEGG_ASCORBATE_AND_ALDARATE_METABOLISM	21	-0.611	-1.972	0	5.69E-03	tags=52%, list=14%, signal=61%
KEGG_ANTIGEN_PROCESSING_AND_PRESENTATION	54	-0.474	-1.905	0	9.80E-03	tags=52%, list=26%, signal=70%
KEGG_SYSTEMIC_LUPUS_ERYTHEMATOSUS	41	-0.490	-1.885	0	1.06E-02	tags=54%, list=26%, signal=72%
KEGG_INTESTINAL_IMMUNE_NETWORK_FOR_IGA_PRODUCTION	28	-0.527	-1.847	4.18E-03	1.20E-02	tags=50%, list=16%, signal=59%
KEGG_ASTHMA	17	-0.604	-1.849	3.93E-03	1.23E-02	tags=76%, list=27%, signal=104%
KEGG_STEROID_HORMONE_BIOSYNTHESIS	40	-0.489	-1.854	0	1.26E-02	tags=43%, list=15%, signal=50%
KEGG_PENTOSE_AND_GLUCURONATE_INTERCONVERSIONS	23	-0.550	-1.826	4.16E-03	1.35E-02	tags=52%, list=14%, signal=61%
KEGG_STARCH_AND_SUCROSE_METABOLISM	35	-0.489	-1.793	6.34E-03	1.64E-02	tags=40%, list=14%, signal=46%
KEGG_GLYCOLYSIS_GLUCONEOGENESIS	51	-0.436	-1.741	4.01E-03	2.41E-02	tags=47%, list=28%, signal=65%
KEGG_CHEMOKINE_SIGNALING_PATHWAY	126	-0.353	-1.694	0	3.35E-02	tags=35%, list=20%, signal=43%
KEGG_RNA_DEGRADATION	51	-0.425	-1.695	2.05E-03	3.50E-02	tags=45%, list=27%, signal=61%
KEGG_PENTOSE_PHOSPHATE_PATHWAY	22	-0.512	-1.673	1.20E-02	3.53E-02	tags=50%, list=19%, signal=61%
KEGG_HEMATOPOIETIC_CELL_LINEAGE	45	-0.435	-1.673	2.02E-03	3.70E-02	tags=31%, list=12%, signal=35%
KEGG_LEISHMANIA_INFECTION	56	-0.401	-1.674	4.06E-03	3.88E-02	tags=54%, list=26%, signal=72%
KEGG_PORPHYRIN_AND_CHLOROPHYLL_METABOLISM	35	-0.442	-1.599	1.23E-02	5.91E-02	tags=43%, list=17%, signal=51%
KEGG_RETINOL_METABOLISM	46	-0.410	-1.599	1.57E-02	6.15E-02	tags=33%, list=12%, signal=37%
KEGG_PROGESTERONE_MEDIATED_OOCYTE_MATURATION	64	-0.376	-1.601	6.56E-03	6.35E-02	tags=44%, list=24%, signal=57%
KEGG_PATHOGENIC_ESCHERICHIA_COLI_INFECTION	46	-0.383	-1.542	2.28E-02	8.06E-02	tags=33%, list=17%, signal=39%
KEGG_NOD_LIKE_RECEPTOR_SIGNALING_PATHWAY	46	-0.391	-1.543	2.77E-02	8.29E-02	tags=33%, list=15%, signal=38%
KEGG_GLYCOSPHINGOLIPID_BIOSYNTHESIS_LACTO_AND_NEOLACTO_SERIES	19	-0.481	-1.547	4.15E-02	8.31E-02	tags=42%, list=21%, signal=53%
KEGG_METABOLISM_OF_XENOBIOTICS_BY_CYTOCHROME_P450	56	-0.371	-1.548	1.23E-02	8.56E-02	tags=30%, list=15%, signal=36%
KEGG_DRUG_METABOLISM_CYTOCHROME_P450	57	-0.368	-1.515	2.79E-02	9.48E-02	tags=28%, list=12%, signal=32%
KEGG_NICOTINATE_AND_NICOTINAMIDE_METABOLISM	17	-0.498	-1.509	5.19E-02	9.60E-02	tags=53%, list=28%, signal=74%
KEGG_FRUCTOSE_AND_MANNOSE_METABOLISM	28	-0.431	-1.495	3.81E-02	1.03E-01	tags=46%, list=24%, signal=61%
KEGG_O_GLYCAN_BIOSYNTHESIS	20	-0.465	-1.479	6.31E-02	1.08E-01	tags=35%, list=10%, signal=39%
KEGG_CELL_CYCLE	113	-0.316	-1.482	8.42E-03	1.09E-01	tags=35%, list=22%, signal=45%
KEGG_ARRHYTHMOGENIC_RIGHT_VENTRICULAR_CARDIOMYOPATHY_ARVC	39	-0.388	-1.469	3.75E-02	1.12E-01	tags=36%, list=23%, signal=46%
KEGG_NATURAL_KILLER_CELL_MEDIATED_CYTOTOXICITY	81	-0.337	-1.462	3.56E-02	1.15E-01	tags=43%, list=27%, signal=59%
KEGG_PROTEASOME	40	-0.382	-1.452	3.48E-02	1.19E-01	tags=35%, list=20%, signal=44%
KEGG_ECM_RECEPTOR_INTERACTION	56	-0.359	-1.433	3.39E-02	1.32E-01	tags=41%, list=23%, signal=53%
KEGG_TYPE_II_DIABETES_MELLITUS	29	-0.398	-1.400	8.25E-02	1.61E-01	tags=66%, list=32%, signal=96%
KEGG_CYTOKINE_CYTOKINE_RECEPTOR_INTERACTION	138	-0.288	-1.385	2.18E-02	1.72E-01	tags=28%, list=19%, signal=34%
KEGG_ARGININE_AND_PROLINE_METABOLISM	45	-0.354	-1.372	5.41E-02	1.81E-01	tags=22%, list=8%, signal=24%
KEGG_GLYCEROLIPID_METABOLISM	30	-0.387	-1.351	1.11E-01	2.03E-01	tags=23%, list=10%, signal=26%
KEGG_CYTOSOLIC_DNA_SENSING_PATHWAY	37	-0.343	-1.315	9.50E-02	2.46E-01	tags=22%, list=10%, signal=24%
KEGG_DILATED_CARDIOMYOPATHY	42	-0.339	-1.305	9.67E-02	2.49E-01	tags=36%, list=23%, signal=46%

FDR, false discovery rate
